# The Effects of Pelvic Floor Muscle Exercise Combined with Core Stability Exercise on Women with Stress Urinary Incontinence following the Treatment of Nonspecific Chronic Low Back Pain

**DOI:** 10.1155/2022/2051374

**Published:** 2022-09-05

**Authors:** Shamima Islam Nipa, Thanyaluck Sriboonreung, Aatit Paungmali, Chailert Phongnarisorn

**Affiliations:** ^1^Department of Physical Therapy, Faculty of Associated Medical Sciences, Chiang Mai University, Mueang, Chiang Mai 50200, Thailand; ^2^Department of Rehabilitation Science, Bangladesh Health Professions Institute (BHPI), Centre for the Rehabilitation of the Paralysed (CRP), Chapain, Savar, Dhaka 1343, Bangladesh; ^3^Department of Obstetrics and Gynecology, Chiang Mai University, Mueang, Chiang Mai 50200, Thailand

## Abstract

**Aim:**

To compare the combined effects of core stability exercise and pelvic floor muscle exercise (PFME) with the effects of PFME alone on women with stress urinary incontinence (SUI) who experience nonspecific chronic low back pain (NSCLBP).

**Methods:**

A stratified randomized controlled trial study (RCT) was conducted with 50 women with SUI who experienced LBP, aged 18–60 years and with pad weight ≥2 grams for the one-hour pad test. The respondents were divided into two groups: the intervention group (PFME + core stability exercise) and the control group (PFME). The primary outcomes were the amount and frequency of urine leakage, which were measured using the one-hour pad test and the Bengali-ISI subjective questionnaire. A secondary outcome was quality of life (QoL), which was measured using King's Health Questionnaire (KHQ). An ITT analysis was conducted using repeated measures ANOVA (2 × 2) with Bonferroni's post-hoc analysis. *Results/Preliminary Findings*. The findings illustrated that 72% (*n* = 18) of the intervention and 28% (*n* = 7) of the control group participants showed improvement in UI after 12 weeks of intervention. In addition, the amount and frequency of urine leakage significantly decreased in the intervention group compared to the control group (*p* ≤ 0.001).

**Conclusion:**

The RCT-illustrated improvement of SUI in women with nonspecific chronic low back pain, reduction of frequency, and improvement of the QoL were more evident from PFME with core stability exercise than from PFME alone.

## 1. Introduction

Urinary incontinence (UI), which occurs more frequently in women, is considered to be one of the major challenges in the 21st century [1]. The International Continence Society (ICS) defines UI as a condition where there is an involuntary loss of urine [2]. Low back pain (LBP) and UI frequently occur simultaneously, and there was a highly clinical association between LBP and UI. Those suffering from LBP are considered to be at more than double the usual risk for UI [2, 3].

Nonspecific low back pain (NSLBP) is commonly found and associated with dysfunction of trunk muscles. Trunk control is relevant with the coordinated activity of muscles of abdominopelvic cavity. Dysfunction of these muscles (pelvic floor muscles, the rectus abdominis, transversus abdominis, and multifidus) might cause spinal instability, pain, and disability [4]. Pelvic floor muscle (PFM) forms the base of core muscles. PFM and core muscles are interdependent with each other [5, 6]. Therefore, chronic low back pain (CLBP) is most often related with weakness of core muscles. The weakness of core muscle might lead to pelvic floor dysfunction resulting UI.

Previous studies have suggested that Pelvic Floor Muscle Exercise (PFME) is effective and works as a first-line treatment approach for intervention in the case of SUI [7]. Pelvic floor muscle contracts in a cranial and forward direction during and prior to physical exertion and high velocity movements including coughing, sneezing, or laughing, thus preventing urine leakage [8].

Core stability exercise is also recommended for the effective management of NSCLBP [4]. Core stability exercise is most frequently used as an intervention of CLBP. Particularly, this exercise helps to stabilize the core muscles along with PFM [9]. The pelvic floor plays a significant role in body's core. At the same time, it contracts the deep abdominal and back muscles. As a result, these muscles provide support, strength, and stability to the spine and internal organs during the movements of the body. Strengthened or strong core muscles provide support the pelvic organs and help to control against leakage. Thus, it can reduce or eliminate UI. Strengthening of the core muscle can reduce the UI, helping to control the leakage [9].

Moreover, there is a high clinical correlation between CLBP and UI. Unfortunately, there is not enough information on effective intervention for UI in the context of NSCLBP. It would be beneficial to determine the therapeutic program and the combination of exercise programs focusing on both UI and NCLBP for the management of the UI condition. Therefore, the aim of the present study is to determine the effects of PFME combined with core stability exercise among women with stress urinary incontinence (SUI).

## 2. Materials and Methods

### 2.1. Study Design and Participants

A stratified randomized controlled trial was conducted with a blinded assessor. The study included 50 women with SUI who experienced LBP. The recruitment process was conducted by two physical therapists. The participants were asked about the presence of involuntary leakage of urine and requested to fill up the Bengali Questionnaire for Urinary Incontinence Diagnosis (QUID) to ensure the presence of SUI. It is a six-items questionnaire. The first 3 items including are summed to determine SUI score. Meanwhile, responses to the rest of the items are used to determine urge urinary incontinence (UUI) score [10]. The more the score, the more the sign of severity of the condition [10]. This was followed by the determination of the inclusion and exclusion criteria. The eligible patients were informed about the objectives of the study and other details surrounding it.

The primary outcome of the study was measuring the amount and frequency of UI among the LBP women. The amount of UI was measured by 1-hour pad test. Pad test is the ICS recommended noninvasive measurement procedure to measure the severity of UI. To conduct the test, at first, patients wore preweighted pad without voiding. Following that, patients were suggested to drink 500 ml of water and take rest for 15 minutes. Subsequently, walk for 30 minutes, one flight stair up and down, 10 times sitting to stand, vigorously cough for 10 times, pick small object from the floor for 5 times, run on the spot for 1 minute and wash hands for 1 minute. After completion of all activities, the pads were removed and reweight the pad to measure amount of urine leakage.

The patients were considered as “incontinent” if the pad weight gained ≥2 grams. [11].

Another primary outcome of the study was to measure the frequency of UI. The Incontinence Severity Index (ISI) is a two-items questionnaire. The first item inquires how often one experiences urinary leakage with four levels of measurement. The second item is used to measure how much urine does one lose each time with three levels of measurement. The total score of the ISI questionnaire is 12, and the more the score, the more the severity [12].

Secondary outcome of the study was measured by the King's Health Questionnaire (KHQ). It is a valid and reliable measurement tool to measure the quality of life (QoL) of women with UI. This questionnaire has three (3) parts including twenty-one (21) items. Part 1 of the questionnaire includes general health perception and incontinence impact (one item each). Part 2 contains role limitations, physical limitations, social limitations (two items each), personal relationships, emotion (three items each) and sleep/energy (two items), and severity measures (four items). Part 3 included single item with ten responses related to frequency, voiding at night, urgency, urge, stress, intercourse incontinence, nocturnal enuresis, infections, pain, and difficulty in voiding. KHQ has four-point rating system. There is 4-point rating system in KHQ, which is scored between 0 and 100. According to the KHQ, the less the score, the better the quality of life; meanwhile, the more the score, the more the sign of severity of the condition [13].

The present study was approved by the Ethical Committee (EC) of the Centre for the Rehabilitation of the Paralysed (CRP), which is organized and operates according to the requirements of the Declaration of Helsinki and International Council for Harmonization-Good Clinical Practice (ICH-GCP) in addition to local regulatory requirements and guidelines. A written consent form was filled up by the patients, who were then randomized into intervention and control groups.

### 2.2. Sample Size Determinations

The sample size estimation was based on the previous study by Burns et al. [14]; the mean frequency of UI episodes between the control group (6.19 episodes per week) and the treatment group (3.3 episodes per week) with a moderate effect size of 0.37 was used in the G∗Power software program for power analysis. The power was set to the level of 0.80, with a *p*-value of 0.05. Therefore, a sample size of 41 participants was required to detect a significant difference between the two groups. To account for the dropout rate, 20% extra participants were added. Therefore, at least 50 participants were required.

### 2.3. Randomization Process

**Figure 1:**
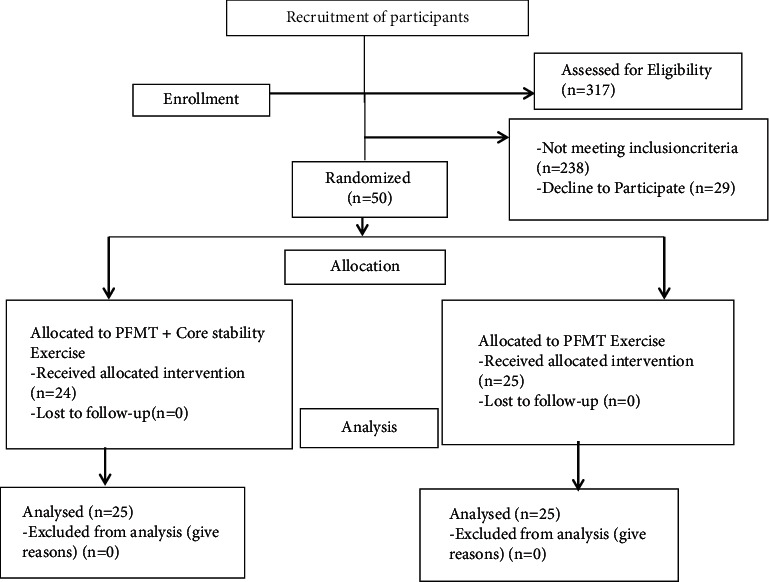
Consolidated Standards of Reporting Trials (CONSORT) flow diagram.

The patients were stratified into two groups according to the age (18–60 years) and the weight of pad test results (2–20 grams of leakage). All the respondents had an equal probability to be sorted into either the intervention group or the control group. This study allocated the respondents with a 1 : 1 parallel ratio with opaque sealed envelopes that were used to achieve the allocation sequence. A researcher who was not involved in the data collection process and treatment created a list of computer-generated random numbers. The participants drew one of the preprinted cards placed in opaque sealed envelopes from boxes labeled “A” (for “core stability exercise with PFME”) and “B” (for “PFME”). The respondents were assigned to the intervention and control groups according to the draw of the card (Figure 1).

### 2.4. Inclusion Criteria

The study included married women aged 18–60 years., with mild-to-moderate severity on the visual analog scale (i.e., VAS 1/10–7/10) of CLBP (>3 months), who had been free from any intervention program for CLBP for at least 1 month. They had to undergo the one-hour pad test with a weight of 2–20 grams and at least have primary education (i.e., can understand and answer the questionnaires). The study included married women who had been pregnant, as pregnancy and childbirth act as significant causal factors for UI.

### 2.5. Exclusion Criteria

The study excluded women who were suffering from musculoskeletal conditions other than NSCLBP, were unable to do the exercise properly, had neurogenic bladder symptoms, were pregnant or in the postpartum period, had urinary tract infection or pelvic floor surgery, and were smokers or alcoholics.

### 2.6. Assessment

The respondents were evaluated during the 1st week, 4th week, 8th week, and 12th week of intervention. They were assessed using the Bengali Incontinence Severity Index (ISI) questionnaire, one-hour pad test, bladder diary, visual analog scale (VAS), pressure biofeedback unit (PBU), and King's Health Questionnaire (KHQ). In addition, before starting the intervention program, the patients of both groups were told the benefits of the intervention and instructed on how to do the exercise.

### 2.7. Training Protocol

The intervention was provided by a well-trained physical therapist. The participants in both groups performed one set of exercises during each week intervention. Besides this, telephone calls, feasible appointments, and counseling of family members were conducted each week to encourage compliance with the intervention.

The primary outcomes were the weight of the pad and the frequency of UI. The degree of UI was measured using the one-hour pad test. If the pad weighed less than 2 grams, the respondent was considered to be cured. Frequency was measured using the Bengali translated and validated ISI questionnaire. The secondary outcome was QoL, which was measured using the Bengali translated KHQ. PBU was used during the 1st session and the 12th week of intervention as an assessment tool to determine the stability of the spinal curve during core muscle activation.

(A) Both groups performed the PFME in supine, sitting, kneeling, and standing positions, which was followed by an increased number of contractions and duration of holding. To ensure that all the participants did the exercises correctly, they were required to meet with the physiotherapist once a month. This research study followed the evidence-based dose of PFME of 8–12 times, 3 times a day. The exercise regimen of holding went from 4 seconds to 30–40 seconds. The contractions were increased up to 20 contractions as progression [15].

(B) The respondents in the intervention group were instructed to go through a very light, low-load core stability consciousness program, including abdominal draw-in and heel slide and heel off exercises, described as follows:

1. To do the core exercises, the participants were first told to lie on the floor. The exercise started with supine lying with knees and hips flexed. Then, the therapist placed 1 or 2 fingers on the abdomen, about 1 to 2 inches inside the hip bones. The respondents were instructed to imagine that they were trying to discontinue urine flow and contract to prevent passing gas. Then, the patients were instructed to slowly drop open the right knee to the right and keep the back and pelvis level. They had to then return to the center and repeat the same action on the left. They were assured that if they did the exercise correctly, they would feel a slight contraction.
2. Next, the patients were instructed to work on the transverse abdominis muscle (core muscle). They were instructed to contract the pelvic floor as above and keep the PFM relaxed. After that, they were suggested to slide the right foot along the floor and straighten the knee. Then, they had to slide the foot back towards the buttock and continue the exercise with the other leg as well [16]. They were told to continue breathing. While breathing out, the patients were instructed to move the lower abdomen up and towards the spine with respect to the navel.
3. Subsequently, the respondents were instructed to lift the right foot 6 inches off from the floor and keep the knees in a bending position. After that, they were asked to bring back the leg. The respondents had to perform the same exercise on the left side as well.

Each item of the exercise was to be repeated for 10 to 20 times on each leg and continued for 2 to 3 sets with 30 to 60 seconds of rest between each set of exercises. The patients were suggested to do the exercises at home for 20 repetitions, two times a day [16].

### 2.8. Statistical Test

Normal distributions of data were assessed using the Shapiro–Wilk test. The effects of treatment and time were examined with the help of a mixed model of two-way repeated measures ANOVA (2 × 2) with Bonferroni post-hoc analysis for all outcome measures. Except for the one-hour pad test, a mixed model of 2 × 4 repeated measures ANOVA for analysis was used at four different time points. All statistical analyses were set at a *p*-value of 0.05 using the SPSS software version 20.0. Data analysis in this study followed the intention-to-treat (ITT) analysis approach.

## 3. Results

There were 50 respondents in the study. Of them, 25 participated in the intervention group and 25 participated in the control group. At the baseline, there were no significant differences between the two groups in terms of demographics and clinical characteristics, which are presented in Table 1.

### 3.1. Primary Outcome Measures

#### 3.1.1. Weight of the Pad

The amount of urine leakage measured by a one-hour pad test was the primary outcome. After 12 weeks of intervention, ITT analysis revealed that the percentage of cured patients (<2 grams) in the intervention and control groups was 72% (*n* = 18/25) and 28% (*n* = 7/25), respectively (Table 2). At baseline, the mean amount of UI of experimental group and control group was 10.36 (SD ± 4.29) and 10.36 (SD ± 3.93), respectively. Notably, after 12 weeks of intervention, the amount of urine leakage in the intervention group was significantly lower (mean 1.36; SD ± 1.09) than in the control group (mean 6.62; SD ± 3.19) (*p*=0.004, Table 3).

#### 3.1.2. Frequency of Urinary Incontinence

The frequency of UI was another important outcome measured by the ISI questionnaire. After 12 weeks of intervention, 14/25 = 56% of the intervention group found themselves “do not leak urine,” whereas only 16% (*n* = 4) of the control group found themselves “do not leak urine.” Furthermore, there was a significant difference in the frequency of urine leakage between the intervention and control groups (*p*=0.014). At baseline, the mean frequency of UI of experimental group and control group was 6.32 (±3.77) and 4.60 (±2.88), respectively. After the 12th week of intervention, the mean frequency of experimental group (0.76; ±1.01) was significantly lower than that of the control group (2.04 ± 1.76) (*p*=0.014, Table 4).

### 3.2. Secondary Outcome of the Study

#### 3.2.1. Quality of Life of Women (QoL) with UI

The KHQ measures QoL, which is the study's secondary outcome. The first part of the questionnaire asked about general health perceptions and the impact of incontinence. After 12 weeks of intervention, 15 out of 25 patients in the intervention group (60%) and 11 out of 25 patients in the control group (44%) said their general health was “very good.” After 12 weeks of intervention, ITT revealed no statistical differences between the experimental group (mean 18; SD ± 18.42) and control group (11 ± 14.57) with level of significance (*p* = 0.291). Furthermore, in the 12th week of intervention, 14 out of 25 patients in the intervention group (56%) and 8 out of 25 patients in the control group (32%) said their bladder problem has “no effect” on their lives. The mean impact on QoL of women in experimental group was 14.52 (±16.71), significantly lower than that of the control group (29.04 ± 23.94) (*p* ≤ 0.001, Table 5).

Role limitation, physical limitations, social limitations, personal relationships, emotions, sleep/energy, and severity measures were all included in Part II of the questionnaire. The present study findings revealed that after the 12th week of intervention, role limitation, physical limitation, and severity measures were significantly improved in the experimental group, 11.30 (±13.34); 11.30 (±12.45); and 31.63 (±8.99), respectively, than in the control group, 17.96 (±15.89); 22.62 (±12.62); and 36.63 (±12.01) (*p* ≤ 0.001 for all variables, Table 5).

The “how much bladder problem” was measured by frequency, nocturia, urgency, urge incontinence, stress incontinence, nocturnal enuresis, intercourse incontinence, and bladder pain in Part III of the questionnaire. After the 12th week of intervention, there was a statistically significant difference between the experimental and control groups [4.40 (±1.41); 7.32 (±1.14)], respectively (*p* ≤ 0.001 for all variables, Table 5).

## 4. Discussion

Both the intervention and control groups showed significant improvements in the primary and secondary clinical efficacy outcomes, according to the study's findings. However, in comparisons between these two types of interventions, the intervention group outperformed the control group on both objective parameters, such as SUI cure and UI frequency reduction.

A previous study found similar results to the current one. That stated that the 12th week abdominal muscle training program is significantly superior to PFM strength training for the treatment of mild SUI among obese patients (*p*=0.005) [17]. In addition, as per the findings of previous study, it was stated that the lower abdominal cavity formed by the PFMs. Therefore, coordinated activity of lower abdominal muscles along with PFMs helps to maintain the UI [18, 19]. The findings of the study are in line with a previous study that indicated the amount of urine leakage significantly decreased in the training group (*p* < 0.05) performing the stabilization exercises [20]. The study also mentioned that the median level of exercise attainment for the exercise group increased significantly from pretest to posttest (*p*=0.01). Consequently, there was a tendency for individuals in the exercise group to perform more complex exercises successfully than those in the non-exercise group (*p*=0.06) [19]. Therefore, it would be considerable to assume that core stability exercise played a significant role in the reduction of UI as the strength and endurance of core muscles were significantly improved in the experimental group.

Conversely, some other studies supported the findings of the control group. There were similar studies stating that PFME was equally effective for both supervised and unsupervised; individual and group training session compared to no treatment [21, 22]. Another study also revealed similar findings to the present study and stated that PFMT increased PFM strength (*p* < 0.05) and significantly reduced the frequency and amount of urine leakage consideringthe level of significance (*p* = 0.04) [19].

In addition, the study's secondary outcome was to use KHQ to assess the QoL of women with UI. After the 12th week of intervention, the researchers discovered a significant difference in between the groups in respect of role play, severity measures, physical limitation, social limitation, and emotion. Because the pelvic floor is an important part of “the powerhouse” for lumbo-pelvic stability, it is possible that PFME helped improve respondents' LBP and QoL. According to a previous study, the mean scores of the domains assessed by KHQ regarding health perception, incontinence impact, daily activity limitations, and severity measures all decreased significantly [11]. Clinical findings from previous studies suggested that the lower abdominal cavity formed by the PFM, as well as the coordinated activity of the lower abdominal muscles in conjunction with the PFM, contributes to the maintenance of UI [16]. At the same time, since the strength and endurance of core muscles were significantly improved in the experimental group, it is reasonable to assume that core stability exercise played a significant role in the reduction of UI. As a result, it would be possible to state that the study findings were not coincidental, but rather clinically significant.

## 5. Conclusion

This study suggests that a program consisting of pelvic floor muscle exercises with low-load core stability consciousness program (abdominal draw-in and heel slide and heel off exercises) may be beneficial for low back pain (LBP) women with stress urinary incontinence (SUI) in reducing the amount (≤2 grams) and frequency of UI, as well as improving QoL outcomes in this population.

## Figures and Tables

**Table 1:** Baseline characteristics of the respondents.

Variables	PFME + core stability group (mean; SD ±)	PFME group (mean; SD ±)	*P*-value^*∗*^
Age (years)	41.00 (±9.07)	40.84 (±8.82)	0.684
BMI (kg/m^2^)	28.90 (±6.81)	27.20 (±6.02)	0.657
Level of exercise	1.96 (±0.84)	1.88 (±0.83)	0.902
Pad test at baseline (g)	10.36 (±4.29)	10.36 (±3.93)	0.513
VAS score (10 mm)	3.44 (±2.00)	3.76 (±1.85)	0.604
ISI frequency	2.68 (±1.10)	2.08(±0.95)	0.144
ISI amount	2.28 (±0.68)	2.08 (±0.49)	0.113
QoL (GH)	45.00 (±31.45)	28.00 (±19.52)	0.092
QoL (II)	49.29 (±16.96)	47.96 (±16.85)	0.618
QoL (RL)	29.29 (±16.87)	28.62 (±17.69)	0.893
QoL (PhyL)	30.62 (±15.73)	32.62 (±12.26)	0.088
QoL (SL)	11.54 (±3.41)	11.98 (±2.94)	0.447
QoL (PR)	21.65 (±10.28)	30.64 (±11.96)	0.311
QoL (EM)	37.74 (±7.01)	27.97 (±7.14)	0.524
QoL (SE)	4.65 (±1.25)	10.64 (±3.46)	0.896

Em: emotion; GH: general health; II: incontinence impact; ISI: incontinence severity index; PBU: pressure biofeedback unit; PhyL: physical limitation; PR: personal relationships; QoL: quality of life; RL: role limitation; SE: sleep energy; SL: social limitation; and SM: severity measure. ^*∗*^Student's *t*-test

**Table 2:** The mean pad weight of the experimental and control groups at each week of intervention.

Measurement week	PFME + core stability mean (±SD) (g); *n*^#^	Mean (±SD) (g); *n*^#^
Pad test at baseline	10.36 (±4.29); *n* = 25	10.36 (±3.93); *n* = 25
Pad test in the 4th week	7.04 (±3.01);	8.66 (±3.51);
	(pad's weight <2 grams; *n* = 8^#^)	(pad's weight<2 grams; *n* = 4^#^)
Pad test in the 8th week	4.06 (±2.10);	8.16 (±3.09);
	(pad's weight <2 grams; *n* = 12^#^)	(pad's weight <2 grams; *n* = 5^#^)
Pad test in the 12th week	1.36 (±1.09);	6.62 (±3.19);
	(pad's weight <2 grams; *n* =18^#^)	(pad's weight <2 grams; *n* = 7^#^)
Cure (%; *n*)	72% (*n* = 18)	28% (*n* = 7)

PFMT: pelvic floor muscle training and SD: standard deviation. *n*^#^: total number of continent patients.

**Table 3:** Difference of pad weight in between the study groups after the 12th week of intervention.

Variable	PFME+ core stability exercise (mean; ±SD) (g)	PFME (mean; ±SD) (g)	*P*-value^*∗∗*^
Pad test weight at baseline	10.36 (±4.29)	10.36 (±3.93)	*p*=0.004^*∗∗*^
Pad test weight after the 12th week of intervention	1.36 (±1.09)^*∗*^	6.62 (±3.19)^*∗*^	

PFMT: pelvic floor muscle training. ^*∗*^Level of significance within the groups *p* < 0.007. ^*∗∗*^Level of significance in between the groups (2 × 2 repeated measures ANOVA test).

**Table 4:** Incontinence severity index after the 12th week of intervention.

Variable	PFME + core stability	PFME	*P*-value
UI frequency at baseline	6.32 (±3.77)	4.60 (±2.88)	0.014^*∗∗*^
UI frequency in the 12th week of intervention	0.76 (±1.01)	2.04 (±1.76)	

PFMT: pelvic floor muscle training. ^*∗*^Level of significance within the groups (*p* < 0.008). ^*∗∗*^Level of significance in between the groups (2 × 2 repeated measures ANOVA test).

**Table 5:** Secondary outcome of the intervention of both groups.

	PFME + core stability exercise (mean; SD ±)	PFME (mean; SD ±)	Level of significance in between the groups *P*^*∗∗*^
Part I			

*General Health Perception*			
Baseline	45 (±31.45)	28 (±19.52)	0.291^*∗∗*^
After the 12th week of intervention	18 (±18.42)^*∗*^	11 (±14.57)^*∗*^	

*Incontinence Impact*			
Baseline	49.29 (±16.96)	47.96 (±16.85)	0.000^*∗∗*^
After the 12th week of intervention	14.52 (±16.71)^*∗*^	29.04 (±23.94)^*∗*^	

Part II			

*Role Limitation*			
Baseline	29.29 (±16.87)	28.62 (±17.69)	0.000^*∗∗*^
In the 12th week of intervention	11.30 (±13.34)^*∗*^	17.96 (±15.89)^*∗*^	

*Physical Limitation*			
Baseline	30.62 (±15.73)	32.62 (±12.26)	0.000^*∗∗*^
12th week of intervention	11.30 (±12.45)^*∗*^	22.62 (±12.62)^*∗*^	

*Social Limitation*			
Baseline	11.54 (±13.39)	11.98 (±11.51)	0.344^*∗∗*^
12th week of intervention	4.88 (±10.17)^*∗*^	3.99 (±8.99)^*∗*^	

*Personal Relationships*			
Baseline	18.65 (±28.98)	30.64 (±25.29)	0.613^*∗∗*^
12th week of intervention	14.64 (±24.19)	25.97 (±21.01)	

*Emotion*			
Baseline	37.74 (±13.21)	27.97 (±15.73)	0.725^*∗∗*^
12th week of intervention	27.08 (±13.99)^*∗*^	16.42 (±12.45)^*∗*^	

*Sleep/Energy*			
Baseline	4.65 (±10.21)	10.64 (±14.32)	0.177^∗∗^
12th week of intervention	3.65 (±8.34)	6.65 (±11.76)	

*Severity Measures*			
Baseline	43.96 (±9.18)	50.64 (±9.60)	0.000^*∗∗*^
12th week of intervention	31.63 (±8.99)^*∗*^	36.63 (±12.01)^*∗*^	

Part III			

How much bladder problem affect?			
Baseline	8.40 (±0.957)	9.36 (±1.11)	0.000^*∗∗*^
12th week of intervention	4.40 (±1.41)^*∗*^	7.32 (±1.14)^*∗*^	

PFMT: pelvic floor muscle training. ^*∗*^Level of significance within the groups (*P* ≤ 0.001). ^*∗∗*^Level of significance in between the groups (2 × 2 repeated measures ANOVA test).

## Data Availability

The interval and ratio data used to support the findings of this study are restricted by the Ethical Review Committee (ERC) of Centre for the Rehabilitation of the Paralysed (CRP), Bangladesh, in order to protect patient privacy. The data are available from the corresponding author for researchers who meet the criteria for access to confidential data.
